# Effects of a combined exercise plus diet program on cardiorespiratory fitness of breast cancer patients

**DOI:** 10.1007/s12282-018-0889-x

**Published:** 2018-07-10

**Authors:** K. Okumatsu, T. Tsujimoto, K. Wakaba, A. Seki, R. Kotake, T. Yamauchi, S. Hirayama, H. Kobayashi, H. Yamauchi, K. Tanaka

**Affiliations:** 1 Graduate School of Comprehensive Human Sciences, Faculty of Health and Sports Sciences, University of Tsukuba, 1-1-1 Tennodai, Tsukuba, Ibaraki 305-8574 Japan; 2 Faculty of Human Sciences, Shimane University, 1060 Nishikawatsucho, Matsue, Shimane 690-8504 Japan; 3 Department of Breast Surgical Oncology, St. Luke’s International Hospital, 9-1 Akashicho, Chou-ku, Tokyo 104-8560 Japan; 4 Division of Medical Oncology, Department of Internal Medicine, St. Luke’s International Hospital, 9-1 Akashicho, Chou-ku, Tokyo 104-8560 Japan; 5 Department of Management and Planning, Central Sports Co., Ltd, 1-21-2 Shinkawa, Chuo-ku, Tokyo 104-8255 Japan; 6 Department of General Medicine, Mito Medical Center, Tsukuba University Hospital, 3-2-7 Miyamachi, Mito, Ibaraki 310-0015 Japan; 7 Faculty of Health and Sports Sciences, University of Tsukuba, 1-1-1 Tennodai, Tsukuba, Ibaraki 305-8574 Japan

**Keywords:** Aerobic fitness, Diet, Weight loss

## Abstract

**Background:**

Decreases in cardiorespiratory fitness among breast cancer patients have often been reported in previous studies, affecting patients’ health and survival. Peak oxygen uptake (${{\dot{V}\text{O}}}_{{{\text{2peak}}}}$) is the gold standard for assessing cardiorespiratory fitness and is inversely correlated with cardiovascular disease among women with breast cancer. Some previous studies have reported that aerobic exercise and proper diet positively influence ${{\dot{V}\text{O}}}_{{{\text{2peak}}}}$. However, almost all studies have been conducted in the Western countries, and few studies are investigating on Asian women who have lower BMI compared with Western ones.

**Purpose:**

Investigating the effects of a combined exercise and diet program among Japanese cancer patients undergoing therapy on ${{\dot{V}\text{O}}}_{{{\text{2peak}}}}$.

**Methods:**

Thirty-two Japanese women with breast cancer undergoing endocrine therapy (age; 50 ± 6 years, body weight; 59 ± 10 kg) were voluntarily assigned to either intervention group (*n* = 21) or control group (*n* = 11). The intervention group completed a 12-week combined exercise plus diet program, consisting of weekly aerobic exercise and maintaining a nutritionally well-balanced 1200 kcal/day diet. The control group was instructed to continue with their usual activities. Anthropometric indices and ${{\dot{V}\text{O}}}_{{{\text{2peak}}}}$ were measured at baseline and after the 12-week program.

**Results:**

All 21 women completed the 12-week program. The ${{\dot{V}\text{O}}}_{{{\text{2peak}}}}$ significantly increased from 26.7 to 30.4 mL/kg/min (1.57–1.62 L/min) in the intervention group, while it remained unchanged (26.9–26.9 mL/kg/min) in the control group. Mean reduction of body mass index was − 2.1 in the intervention group (*P* < .001) and + 0.1 in the control group.

**Conclusions:**

Our combined exercise plus diet program may contribute to improvement in cardiorespiratory fitness and body weight compared with control group.

## Introduction

Breast cancer is the most common cancer and the leading causes of cancer death among women worldwide [1]. According to the previous study, adjuvant breast cancer therapies (e.g., chemotherapy, endocrine therapy) associate with cardiotoxicity and cardiovascular disease (CVD) [2]. These treatments reduce cardiorespiratory fitness (CRF). Peak oxygen intake (${{\dot{V}\text{O}}}_{{{\text{2peak}}}}$), as the gold standard measurement of cardiorespiratory fitness, is decreased by 6–10% among breast cancer patients relative to healthy individuals [3–5]. The poor CRF can adversely affect the quality of life as CRF strongly relates to daily activities [5]. Various studies recommend that exercise can improve the cardiorespiratory fitness among CVD patients and breast cancer survivors [6–8]. However, regarding breast cancer, few studies have investigated the effect of combined exercise and diet program on ${{\dot{V}\text{O}}}_{{{\text{2peak}}}}$ in the world. Regarding different race, food style, exercise habit, there are large differences between Western countries and Asian countries. In Asia, however, there is no research which focused on this point. The purpose of this study is to investigate the effects of exercise plus diet program on cardiorespiratory fitness among Japanese breast cancer patients. We hypothesized that the combined exercise and diet program would have significantly improved 3.0 to 5.0 mL/kg/min of ${{\dot{V}\text{O}}}_{{{\text{2peak}}}}$ compared to the usual-care group at 3 months.

## Patients and methods

### Participants and randomization

We recruited participants from August 27, 2016, through September 6, 2016, from St. Luke International Hospital in Tokyo, Japan. Thirty-two breast cancer patients undergoing an endocrine therapy were registered in this study. The inclusion criteria in this study were as follows: (1) female survivor at least 1 year after surgery; (2) age between 20 and 74 years old; (3) receiving an aromatase inhibitor or a tamoxifen at least 1 year; (4) no medical conditions or contraindicated medication that would prohibit participation in our exercise plus diet program; (5) experiencing various side effects after taking an endocrine therapy (weight gain, cancer-related fatigue and, deterioration of the quality of life, and so on).

Study participants were non-randomly assigned to a combined exercise plus diet group (intervention group) or usual-care group based on their desirability. The Ethical Committee of the both University of Tsukuba and the St. Luke International Hospital approved the study protocol. The protocol has been registered with the UMIN Clinical Trials Registry (UMIN000025890). This study was conducted in accordance with the guidelines proposed in the Declaration of Helsinki. All participants have provided written informed consent documents and permission from a physician before participating in any study-related activities.

### Dietary intervention

All participants were instructed to restrict their energy intake to approximately 1200 kcal/day. The diet program is based on the Four-Group Point Method which divides foods into four groups to calculate energy intake and nutrient balance easily [9]. Each food groups (FG) is based on their nutrient contents. The energy intake per meal (3 times per day) was as follows: 80 kcal from FG 1 (eggs, dairy products); 80 kcal from FG 2 (meat, fish, and soybean products); 80 kcal from FG 3 (vegetables and fruits); and 160 kcal from FG 4 (carbohydrates and oils). The participants maintained a daily food diary during the 12-week program and attended weekly group-based 90-min lectures on well-balanced diets where dieticians encouraged weight loss through nutritional education and dietary behavior modification.

Our research group has conducted weight loss studies for more than 30 years, and our weight loss program is already applied to health promotion activities in the surrounding communities [10]. We confirmed that our program is safe and sufficiently effective in reducing body weight while maintaining participant’s nutritional balance [11, 12]. In this study, we consulted with breast cancer specialist and adopted our established dietary program in consideration of its safety and effectiveness. A more detailed explanation of the program and methodology has been previously published [12].

### Exercise intervention

The 3-month exercise intervention was a combination of aerobic exercise and resistance exercise once per week (45 min per lecture, 2 times in every 12 sessions). Japanese-certified exercise trainer supervised both exercise programs at a Central Sports Fitness Club in Tokyo, Japan. After each exercise session, every participant recorded the type, duration, and intensity as a measurement of exercise adherence. Every participant in the exercise group turned in exercise records to the fitness trainers at the beginning of each exercise session, and the stuff recorded attendance of participants. The aerobic exercise consisted of primarily of aerobic dance, yoga, and brisk walking although, participants could choose another aerobic exercise, such as stationary cycling and walking in the pool. The resistance exercise mainly consisted of six exercises (e.g., chest press, lat pull down, seated row, leg press, leg curl, and leg extension) which were performed for 8–20 repetitions for 2–3 sets. During resistance exercise session, the stuff divided participants into a small group of 5–7 people to supervise them carefully. Before 4 weeks, exercise intensity (rated perceived exertion: RPE) was maintained at low to moderate, i.e., 7–12, and after 4 weeks, the exercise intensity was increased up to 10–15. The exercise professionals systematically asked all participants about any physical conditions each week to identify any changes in participants. Before this intervention, we discussed content of intervention with physiotherapists and physician of breast cancer to create this exercise program.

### Measurements

#### Anthropometry and body composition

Height was measured to the nearest 0.1 cm using a wall-mounted stadiometer (YG-200; Yagami, Japan), and weight was measured to the nearest 0.05 kg using a digital scale (TBF-551; Tanita, Japan). For weight measurement, the participants were in their underwear, and they did not wear shoes. Body mass index was calculated as weight (kilograms) divided by height (in meters) squared. We assessed the percentage of body fat using a bioelectrical impedance device which gives off 50 kHz, standing foot to foot, and body fat was calculated using the manufacturer’s algorithm (MC-190; Tanita, Japan). Abdominal circumference was measured to the nearest 0.1 cm at the level of the umbilicus using a flexible, retractable, fiberglass tape measure.

#### Sociodemographic and lifestyle variables

Participants also reported sociodemographic and lifestyle characteristics via self-administered questionnaires at baseline. Characteristics included education status (whether a college graduate or not), time (year) since cancer diagnosis, and current smoking and drinking habits (yes or no for both). Participants answered their cancer stage and type of treatment.

#### Blood pressure

Blood pressure was measured using an automated sphygmomanometer (HEM-7511T; Omron Healthcare, Japan) in the seated position after a 5-min rest period. The average value of the two readings was used for data analysis.

#### Energy intake

All food and beverages intake, in kilocalories, and macronutrient intake, in grams, were assessed by 3-day weighed food records. Participants recorded everything they consumed for 3 days, including 2 weekdays and 1 weekend day before and during a 2-week period (weeks 11 and 12) of the intervention program. Daily energy intake and macronutrient composition were assessed using commercially available software (Excel Eiyo-Kun; Kenpakusya, Japan) by a skilled nutritionist.

#### Aerobic capacity (maximum progressive exercise testing)

The participants performed an incremental exercise test using a cycling ergometer (828E; Monark, Sweden) to determine their peak oxygen intake (${{\dot{V}\text{O}}}_{{{\text{2peak}}}}$). Following a 2-min warm-up at 15 or 30 W, the workload increased every minute by 15 W until volitional exhaustion. Participants cycled at a cadence of 60 rpm. During the test, heart rate, RPE were measured by trained staff. ${{\dot{V}\text{O}}}_{{{\text{2peak}}}}$ was calculated using developed prediction equation by Okura et al. [13]. This equation consisted of the following independent variables work rate divided by body weight, age, and percentage body fat, which significantly correlated with measured ${{\dot{V}\text{O}}}_{{{\text{2peak}}}}$. Reliability of this prediction has already proved (*n* = 83, *r* = 0.83, standard error of the estimate = 3.66 mL/kg/min), and a more detailed description of the methodology has been previously published [13].

### Statistical analysis

Values are expressed as the mean ± standard deviation. Paired Student’s *t* test was performed to test the significance of changes in values. Categorical variables were compared between the two study groups using a Chi-squared test. Unpaired *t* test was used to compare values between two groups. To compare any change in each item between groups, a two-way repeated measures analysis of variance (time × group) was applied. *P* value < 0.05 was considered statistically significant. Our primary analysis was based on an intention treat-to-treat (ITT) principle, with missing data replaced by baseline observations carried forward. We performed all statistical analysis using SPSS ver 21.

## Results

**Fig. 1:**
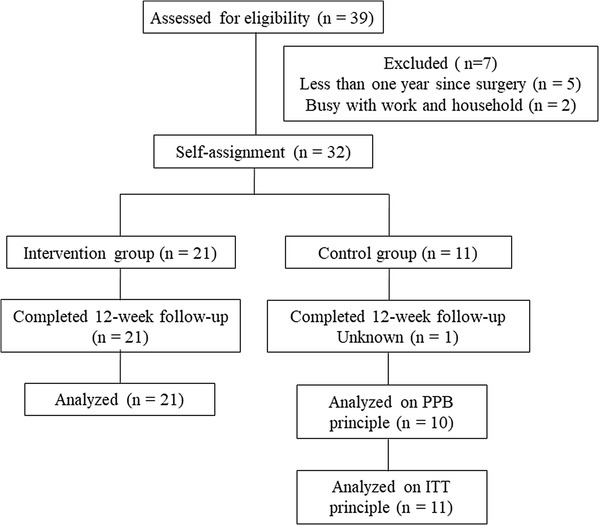
Flow diagram of participants through the 12-week non-randomized trial

The complete flow diagram of the study participants is presented in Fig. 1. We faced unexpected difficulties in recruiting participants and eventually invited 39 candidates to the orientation session. Before baseline examination, seven candidates are excluded due to the criteria or busy work and household. Thirty-two participants met the eligibility criteria and non-randomly assigned to the intervention group (*n* = 21) and usual-care group (*n* = 11). The 32 participants were subject to the ITT analysis.

### Characteristics

Table 1 shows characteristics of the participants. Fifty-nine percent of cancer stage was I or II and the average time since diagnosis was 3.5 years. The mean chronological age was 54 ± 5 years old (range 42–64 years). There was no significant difference in cancer stage, cancer treatment, and smoking habit between intervention group and usual-care group except for education history.

**Table 1:** Baseline characteristics of study participants

Characteristics	Intervention group (*n* = 21)	Usual-care group (*n* = 11)	*P*
Age, years (mean ± SD)	51 ± 6	53 ± 5	0.14
Race, no. (%)			
Asian (Japanese)	21 (100)	11 (100)	
Education, no. (%)			0.04
High school	6 (29)	0	
Some college	12 (57)	5 (45)	
≧ College	3 (14)	6 (55)	
Time since cancer diagnosis, years (mean ± SD)	4 ± 2	3 ± 1	0.14
Disease stage, no. (%)			0.71
0	1 (5)	0 (0)	
I	10 (48)	8 (73)	
II	7 (33)	2 (18)	
III	3 (14)	1 (9)	
Postmenopausal at baseline, (%)	7 (33)	4 (11)	0.62
Cancer treatment, no. (%)			
Surgery	21 (100)	11 (100)	–
Chemotherapy	8 (38)	3 (27)	0.31
Radiation	13 (62)	8 (73)	0.66
Tamoxifen	14 (67)	7 (64)	0.14
Aromatase inhibitor	7 (33)	4 (36)	–
Smoking habit, no. (%)			0.68
Never	1 (5)	0 (0)	
Former	4 (19)	3 (27)	
Current	16 (76)	8 (73)	

SD, standard deviation

### Changes in body composition

Table 2 shows the changes in body composition in both groups. Regarding body weight, body mass index, percent body fat, and abdominal circumference, there were significant time-by-group interactions (*p* < 0.001).

**Table 2:** Characteristics of the participants before and after the program

Measures	Intervention group (*n* = 21)		Usual-care group (*n* = 11)		*P* for interaction
	Mean	SD	Mean	SD	
Body weight, kg					< 0.001
Baseline	60.2	10.8	54.1	8.0	
12 weeks	54.7	9.9*	53.9	7.9	
Body mass index, kg/m^2^					< 0.001
Baseline	24.3	4.2	21.9	3.8	
12 weeks	22.2	3.9*	21.9	3.5	
Body fat, %					< 0.001
Baseline	33.5	7.7	30.4	7.2	
12 weeks	29.1	8.0*	30.4	6.5	
Abdominal circumference, cm					< 0.001
Baseline	86.5	11.2	81.2	7.4	
12 weeks	79.9	12.0*	82.7	5.8	
Systolic blood pressure (mmHg)					0.175
Baseline	120.5	21.3	119.6	10.9	
12 weeks	114.5	17.6*	118.2	9.8	
Diastolic blood pressure (mmHg)					0.527
Baseline	81.4	11.2	81.7	8.3	
12 weeks	80.0	9.7*	79.6	8.6	
${{\dot{V}\text{O}}}_{{{\text{2peak}}}}$, mL/kg per minute					0.002
Baseline	26.7	4.6	26.9	5.0	
12 weeks	30.4	5.4*	26.9	5.1	
${{\dot{V}\text{O}}}_{{{\text{2peak}}}}$, mL per minute					
Baseline	1569	171	1467	259	0.244
12 weeks	1622	139*	1458	213*	
Weight gain since cancer diagnosis, kg	4.8	3.6	2.0	5.0	0.180
Weekly exercise session attendance, %	88.1	7.1	NA		
Weekly diet session attendance, %	81.0	11.8	NA		

SD, standard deviation; ${{\dot{V}\text{O}}}_{{{\text{2peak}}}}$, peak oxygen consumption. *Significant change within group by paired *t* test (*P* < 0.05)

### Adherence to the interventions

Adeherence of intervention before and after the program is also shown in Table 2. Adherence to the exercise program averaged 88% (range 76–95%) and diet program’s adherence averaged 81% (range 67–100%) in the intervention group. All participants in the intervention group completed the 12-week intervention (no dropouts). Regarding control group, 1 participant dropped out during this study.

### Changes in dietary intake

As shown in Table 3, in the intervention group, the mean total energy intake calculated from the available food records significantly decreased from 1765 ± 289 to 1461 ± 316 kcal. Fat and carbohydrate intake also decreased significantly in the intervention group (Table 3). No adverse event was reported in the intervention group.

**Table 3:** Change in dietary intake during 3-month intervention

	Intervention group			Control group			*P* value for	
	Baseline	12 weeks	Change	Baseline	12 weeks	Change	Group difference at baseline	*p* for interaction
Dietary intake, kcal/day	1765 ± 289	1461 ± 316	− 303 (− 491 to − 116)	1618 ± 222	1578 ± 302	− 40 (− 330 to 251)	0.242	0.101
Protein intake, g/day	66 ± 15	63 ± 15	− 4 (− 12 to 4)	67 ± 15	63 ± 17	− 4 (− 16 to 8)	0.766	0.963
Fat intake, g/day	62 ± 13	52 ± 11	− 10 (− 18 to − 2)	52 ± 9	51 ± 16	− 1 (− 14 to 12)	0.050	0.197
Carbohydrate intake, g/day	220 ± 50	180 ± 53	− 40 (− 70 to − 9)	217 ± 30	213 ± 41	− 4 (− 44 to 34)	0.984	0.151

Values are presented as mean ± standard deviation. Changes from baseline are presented as mean (95% confidence interval)

### Changes in cardiorespiratory fitness

**Fig. 2:**
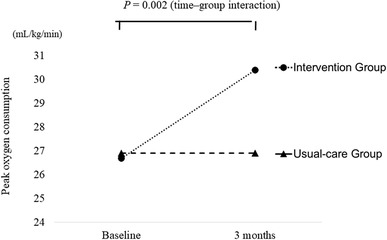
Changes in ${{\dot{V}\text{O}}}_{{{\text{2peak}}}}$ during entire study period by group

Figure 2 presents changes in ${{\dot{V}\text{O}}}_{{{\text{2peak}}}}$ during the entire study period by the group. At the baseline, ${{\dot{V}\text{O}}}_{{{\text{2peak}}}}$ of the intervention group was 26.7 ± 4.6, and usual-care’s ${{\dot{V}\text{O}}}_{{{\text{2peak}}}}$ was 26.9 ± 5.0. After the intervention, the ${{\dot{V}\text{O}}}_{{{\text{2peak}}}}$ of the intervention group was 30.4 ± 5.4, and usual-care’s value was 26.9 ± 5.1. No significant ${{\dot{V}\text{O}}}_{{{\text{2peak}}}}$ difference was observed between the two groups at baseline. However, significant increases in ${{\dot{V}\text{O}}}_{{{\text{2peak}}}}$ were found in the intervention group after the 3 months. During this period, a significant interaction (time × group) was observed between the two groups (*p* = 0.002).

## Discussion

We hypothesized that the combined exercise and diet program would have significantly improved around 3.0–5.0 mL/kg/min of ${{\dot{V}\text{O}}}_{{{\text{2peak}}}}$ compared to the usual-care group at 3 months. We have demonstrated that this program significantly improved 3.7 mL/kg/min of ${{\dot{V}\text{O}}}_{{{\text{2peak}}}}$ compared with usual-care group. As a result, this program supports the hypothesis.

Through this study, our results suggested two points. (1) Reduction of body weight, percent body fat and, abdominal circumference was significantly greater in the intervention group compared with the usual-care group; (2) ${{\dot{V}\text{O}}}_{{{\text{2peak}}}}$ was significantly improved in the intervention group than the usual-care group.

Our first results suggested that combined exercise plus diet program may be more effective for weight loss than usual-care. There was a significant decrease in body mass index in the intervention group (− 2.1 ± 0.9) during the 12-week intervention, but there was no significant reduction in the control group (0.1 ± 0.5). Mefferd et al. investigated the effect of exercise plus dietary intervention on body mass index among breast cancer survivors, stating that exercise plus dietary intervention group decreases their body mass index significantly during 16 weeks, but there was no significant reduction in the control group [14]. Our study supports this result, and a combined exercise plus diet program may reduce more body mass index compared with usual-care among breast cancer survivors.

Our second results suggested that combined exercise plus diet program was effective to improve cardiorespiratory fitness than control group. First, calculating ${{\dot{V}\text{O}}}_{{{\text{2peak}}}}$ is related to body weight, and the participants in intervention group reduced their body weight largely so, weight loss contributed increasing ${{\dot{V}\text{O}}}_{{{\text{2peak}}}}$. However, participants increased not only relative ${{\dot{V}\text{O}}}_{{{\text{2peak}}}}$ (mL/min/kg), but also absolute ${{\dot{V}\text{O}}}_{{{\text{2peak}}}}$ (mL/min). Through our exercise intervention, participants did aerobic exercise regularly, so they could increase cardiorespiratory fitness. This result suggests that combined exercise plus diet program is a useful method to keep or increase cardiorespiratory fitness with weight loss. The previous meta-analysis also reported that almost all study which conducts the aerobic exercise has a positive effect for the breast cancer patients during treatment [15].

The limitations of this study are as follows. First, the relatively small number of participants may increase the probability of type 2 errors. Second, the study was 12 weeks in duration, which was a rather short period for observing ${{\dot{V}\text{O}}}_{{{\text{2peak}}}}$. With a longer observation period for the weight loss program, we would be in a better position to comment on the long-term effects of combined exercise plus diet program. Third, study participants were non-randomly assigned to a combined exercise plus diet group or usual-care group based on their desirability. This non-random assignment would lead to biased results. For instance, those people who would like to participate actively in this intervention program maybe tend to have a healthier lifestyle and/or tend to take care of their own body condition compared to those who would not like to participate in this study. Fourth, physiological function might be different according to the age and/or the menopausal status of subjects. Due to the light of sample size, we could not discuss about the influence of menopausal status on breast cancer patients. Finally, we did not assign participants to only exercise or only diet group; so we cannot conclude that whether exercise or diet program is more effective to improve the cardiorespiratory fitness.

In conclusion, the results of this study demonstrated that our combined exercise plus diet program increased cardiorespiratory fitness and decreased body weight, percent body fat, and body mass index. A further detailed study involving a large sample of exercise or diet group is required to demonstrate the effectiveness in improving cardiorespiratory fitness accompanying weight loss. We need to follow a longer term to investigate the effects of exercise and diet program for breast cancer patients in the future.
